# The *superwoman1‐cleistogamy2* mutant is a novel resource for gene containment in rice

**DOI:** 10.1111/pbi.12594

**Published:** 2016-07-18

**Authors:** Fabien Lombardo, Makoto Kuroki, Shan‐Guo Yao, Hiroyuki Shimizu, Tomohito Ikegaya, Mayumi Kimizu, Shinnosuke Ohmori, Takashi Akiyama, Takami Hayashi, Tomoya Yamaguchi, Setsuo Koike, Osamu Yatou, Hitoshi Yoshida

**Affiliations:** ^1^ Division of Applied GeneticsInstitute of Agrobiological SciencesNational Agriculture and Food Research Organization (NARO)IbarakiJapan; ^2^ Division of Crop Breeding ResearchHokkaido Agricultural Research CenterNAROHokkaidoJapan; ^3^ Division of Rice ResearchInstitute of Crop ScienceNAROIbarakiJapan; ^4^ Division of Crop DevelopmentCentral Region Agricultural Research CenterNARONiigataJapan; ^5^ Division of Agro‐Production Technologies and Management ResearchTohoku Agricultural Research CenterNAROIwateJapan; ^6^ Present address: Center for Genome BiologyInstitute of Genetics and Developmental BiologyChinese Academy of SciencesBeijing100101China; ^7^ Present address: Agriculture, Forestry and Fisheries Research CouncilMinistry of Agriculture, Forestry and Fisheries of JapanTokyo100‐8950Japan

**Keywords:** cleistogamy, MADS‐box gene, breeding, GMO, gene flow, lodicule

## Abstract

Outcrossing between cultivated plants and their related wild species may result in the loss of favourable agricultural traits in the progeny or escape of transgenes in the environment. Outcrossing can be physically prevented by using cleistogamous (i.e. closed‐flower) plants. In rice, flower opening is dependent on the mechanical action of fleshy organs called lodicules, which are generally regarded as the grass petal equivalents. Lodicule identity and development are specified by the action of protein complexes involving the SPW1 and OsMADS2 transcription factors. In the *superwoman1‐cleistogamy1* (*spw1‐cls1*) mutant, SPW1 is impaired for heterodimerization with OsMADS2 and consequently *spw1‐cls1* shows thin, ineffective lodicules. However, low temperatures help stabilise the mutated SPW1/OsMADS2 heterodimer and lodicule development is restored when *spw1‐cls1* is grown in a cold environment, resulting in the loss of the cleistogamous phenotype. To identify a novel, temperature‐stable cleistogamous allele of *SPW1*, targeted and random mutations were introduced into the *SPW1* sequence and their effects over SPW1/OsMADS2 dimer formation were assessed in yeast two‐hybrid experiments. In parallel, a novel cleistogamous allele of *SPW*1 called *spw1‐cls2* was isolated from a forward genetic screen. In *spw1‐cls2*, a mutation leading to a change of an amino acid involved in DNA binding by the transcription factor was identified. Fertility of *spw1‐cls2* is somewhat decreased under low temperatures but unlike for *spw1‐cls1*, the cleistogamous phenotype is maintained, making the line a safer and valuable genetic resource for gene containment.

## Introduction

In rice (*Oryza sativa* L.) stigmas and pollen mature before flower opening, thereby promoting self‐pollination. Outcrossing rates in cultivated rice are estimated to be less than one per cent (Messeguer *et al*., 2001); however, figures well above twenty per cent have been reported for crosses between cultivated and wild rice species when grown in specific conditions (Marathi and Jena, 2014; Phan *et al*., 2012). Cross‐pollination between cultivated species and weedy wild relatives is problematic to rice producers as it may hinder the effectiveness of weed management and/or lead to the loss of favourable traits in a given line (Gealy *et al*., 2015). For example, Clearfield^®^ rice is a commercial, nontransgenic variety of rice which is resistant to a herbicide called imazethapyr. The herbicide has been used efficiently to weed out red rice, an invasive wild relative, from fields cultivated with Clearfield^®^ rice. However, outcrosses between Clearfield^®^ rice and weedy red rice in some areas have resulted in an imazethapyr‐resistant hybrid rice, making the application of the herbicide inefficient (Sudianto *et al*., 2013). The eventuality of such ‘gene flow’ from cultivated species to their wild relatives, and most particularly in case of genetically modified (GM) crops, is a cause of concern and has called for the development of so‐called gene containment methods (Daniell, 2002; Gressel, 2014). Various strategies have been designed, such as transgene removal from pollen by molecular excision, transgene splitting using intein flanking sequences, male sterility or generation of cleistogamous (i.e. self‐pollinating, closed‐flower) plants to prevent pollen dispersal (Moon *et al*., 2011; Ohmori *et al*., 2012; Shinoyama *et al*., 2011; Toppino *et al*., 2011; Wang *et al*., 2014). In rice, the cleistogamy trait can be introduced by modification of a single gene. In contrast to most other strategies, engineering cleistogamy in rice is relatively straightforward and does not necessarily require the introduction of several transgenes, as described hereafter.

Rice flowers bear specialised perianth structures, the lemma and palea, which tightly enclose the inner sexual organs. At anthesis, the flower opens under the mechanical pressure exerted by the lodicules, two fleshy organs which swell and push the lemma and palea open to allow anther exertion and pollen dispersal. Cleistogamy can be induced by altering lodicule morphology so that the organs are unable to exert sufficient outward pressure to trigger flower opening, such as in the *spw1‐cls* mutant (Yoshida *et al*., 2007). The pollen then remains entrapped within the closed flower and no outcrossing can occur (Ohmori *et al*., 2012).

**Figure 1:**
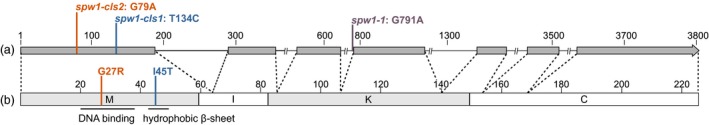
Representation of the *SPW1* gene (a) and its product (b). a: Exons are represented with grey boxes and introns with black solid lines. Domains of the SPW1 protein are abbreviated as follows: M for MADS, I for intervening, K for keratin‐like and C for C‐terminal. b: Positions of the highly conserved region critical for DNA binding and the β‐sheet involved in dimerisation mutated in *spw1‐cls2* and *spw1‐cls1*, respectively, are underlined.

At the molecular level, rice lodicule identity is mainly specified by the action of two transcription factors, SUPERWOMAN1 (SPW1) and OsMADS2. Both proteins are encoded by MIKC‐type MADS‐box genes and consist of a highly conserved N‐terminal DNA‐binding domain (MADS domain) followed by two domains involved in protein–protein interactions (Intervening and, predominantly, Keratin‐like domain) and a C‐terminal domain supporting different functions, notably transcriptional control (Kaufmann *et al*., 2005). Upon heterodimerization, SPW1 and OsMADS2 are thought to bind to target DNA motifs called CArG boxes and regulate gene expression (Shore and Sharrocks, 1995; Yao *et al*., 2008), although it is likely that the heterodimer functions within larger protein complexes (Theissen and Saedler, 2001). In addition to specifying lodicule identity, SPW1 and OsMADS2 are also driving stamen development in the third floral whorl (Yoshida and Nagato, 2011). No *osmads2* mutant has been isolated so far, in contrast several *spw1* mutants have been described in the literature. In an *spw1* loss‐of‐function mutant, such as in the *spw1‐1* mutant, lodicules are homeotically transformed into glume‐like organs and male organs (i.e. stamen) into female (i.e. carpel‐like) organs, hence the *superwoman1* gene designation (Nagasawa *et al*., 2003). Over‐expression of the *SPW1* gene results in a phenotype opposite to that of *spw1*, that is a female to male (i.e. carpel to stamen‐like) organ transformation, a phenotype also referred to as ‘superman’ (Lee *et al*., 2003). In the weak *superwoman1‐cleistogamy* (*spw1‐cls*) allele, the isoleucine in position 45 of the SPW1 protein is replaced by a threonine (I45T; Yoshida *et al*., 2007). The amino acid I45 is part of a β‐strand within the dimerisation interface consisting of predominantly hydrophobic amino acids (Figure 1; Shore and Sharrocks, 1995). The I45T change lowers the hydrophobicity of the region and disturbs SPW1/OsMADS2 dimerisation. Consequently, lodicules are elongated in *spw1‐cls* and flowers are unable to open. Although stamen formation is also dependent on the SPW1/OsMADS2 dimer activity, the stamens of *spw1‐cls* show no visible defects and the plants are fertile. Most likely, the remaining biological activity of the SPW1^I45T^/OsMADS2 heterodimer is sufficient to support proper stamen development in *spw1‐cls* (Yoshida, 2012; Yoshida *et al*., 2007). Furthermore, SPW1 can also dimerise with OsMADS4, which is encoded by a paralog of *OsMADS2* mainly expressed in the third whorl, and the SPW1^I45T^/OsMADS4 dimer is also expected to contribute to stamen development in *spw1‐cls* (Yao *et al*., 2008).

The single‐locus, non‐transgenic nature of the cleistogamy trait in *spw1‐cls* makes the line attractive for gene containment purposes; however, extensive studies have revealed that when plants are grown in a cold environment the SPW1^I45T^/OsMADS2 dimer regains enough stability to drive substantial lodicule development and consequently the cleistogamous phenotype of *spw1‐cls* is lost (Yoshida *et al*., 2007; SO and HY, unpublished data).

In the present work, it was set out to identify mutants that show substantial lodicule elongation, ensuring a stable cleistogamy even in a cold environment, but also normal stamen development to maintain satisfactory fertility rates. The possibility of fine‐tuning the biological activity of the SPW1/OsMADS2 heterodimer was investigated in a reverse genetic approach. Dimer formation between OsMADS2 and directly and randomly mutated versions of the SPW1 protein was assessed in yeast two‐hybrid experiments. Subsequently, transgenic lines were generated using selected mutant constructs and their phenotypes were confirmed *in planta*.

Concomitantly, a chemically mutagenised population of rice was screened in a forward genetic approach to allow for the identification of cold‐stable cleistogamous lines not restricted to heterodimerization mutants. The isolation of a novel cleistogamous allele of *SPW1*, called *spw1‐cls2*, is described. The mutation in *spw1‐cls2* leads to a change of an amino acid involved in DNA binding and, unlike for *spw1‐cls*, cleistogamy is maintained when plants are grown in a cold environment. The present work shows that the *spw1‐cls2* line can be used advantageously in gene containment strategies.

## Results

### Identification of mutated versions of the SPW1 protein impaired for heterodimerization with OsMADS2

With the recent techniques collectively referred to as genome editing, directed mutagenesis in a plant genome is becoming possible (Osakabe and Osakabe, 2015; Schaeffer and Nakata, 2015). In this context, it was set out to confirm in an initial study that lodicule development could be manipulated by introducing various mutations in the *SPW1* sequence without compromising plant fertility. To identify mutations causing intermediate destabilisations of the SPW1/OsMADS2 heterodimer, the SPW1 protein was modified at specific amino acid positions and dimerisation with OsMADS2 was estimated for each generated mutant using a yeast two‐hybrid colorimetric filter assay. In *spw1‐cls*, the mutated amino acid (I45) belongs to a hydrophobic β‐strand involved in dimerisation with OsMADS2 and thus mutations at this position were initially favoured in the experimental design. A total of 11 cDNAs encoding for amino acids with gradually decreasing hydrophobicity indices were designed, including amino acids with charged (I45D, I45R), aromatic (I45Y) and unique (I45G) side chains. The binding affinities between OsMADS2 and each of the mutated SPW1 proteins were estimated from the resulting colouring intensities in the filter assay. It was hoped to identify mutations slightly more severe than in *spw1‐cls*, which would correspond to coloration intensities somewhat weaker than the one obtained for the SPW1^I45T^ control. In our experimental conditions, filter spots corresponding to the SPW1^WT^, SPW1^I45T^ and empty vector controls showed a strong, fair or no coloration, respectively (Table 1). Substitution with amino acids with hydrophilic (S, Q, R, N, D) or neutral (G) side chains all resulted in a faint colouring, indicative of a very low binding affinity between the two proteins. For amino acids with hydrophobic side chains excepted cysteine (i.e. F, M, Y, A), colouring intensities were comparable or stronger than the one of the SPW1^I45T^ control. Substitution with cysteine (C) also resulted in a faint colouring. These results indicate that up to now the investigated mutations had either too weak or too strong effects over SPW1/OsMADS2 dimerisation to be considered candidates for a prospective genome editing.

**Table 1:** Colouring intensities obtained for various mutants of *spw1* in a colorimetric assay

	Amino acid in position 45	Colouring intensity	Hydrophobicity index
Controls	Isoleucine (I; WT)	+++++	99
	Threonine (T; *cls1*)	++++	13
	Null (empty vector)	−	n.a.
Mutants via directed mutagenesis	Phenylalanine (F)	++++	100
	Methionine (M)	+++++	74
	Tyrosine (Y)	++++	63
	Cysteine (C)	+	49
	Alanine (A)	++++	41
	Glycine (G)	+	0
	Serine (S)	+	−5
	Glutamine (Q)	+	−10
	Arginine (R)	+	−14
	Asparagine (N)	+	−28
	Aspartic acid (D)	+	−55

Colouring intensities reflect the binding affinities between OsMADS2 and each mutated SPW1 protein in a yeast two‐hybrid filter assay. Hydrophobicity indices are given for pH = 7 (Monera *et al*., 1995).

To test for mutations outside the 45th position of the SPW1 protein, a random mutagenesis strategy was followed and a yeast two‐hybrid library of *SPW1* mutant cDNAs was created using error‐prone PCR amplification. About 10 000 independent clones were screened in a colorimetric assay. This time, several clones showing intermediate colouring intensities were isolated and, after identification of the mutated loci by sequencing, nine clones were eventually selected for further analysis. Among these, five clones carried two mutations affecting different domains of the SPW1 protein: one clone was mutated for the MADS domain (E34G/I46V), two clones for both the MADS and the K domains (E34G/E82G; T51A/L79P), and two other clones for both the K and the C‐terminal domains (N98D/A131V; G110R/T144I). The remaining four clones each carried a single mutation affecting the MADS domain (L35F; F57S), the I domain (I67T), or the K domain (W80R).

**Figure 2:**
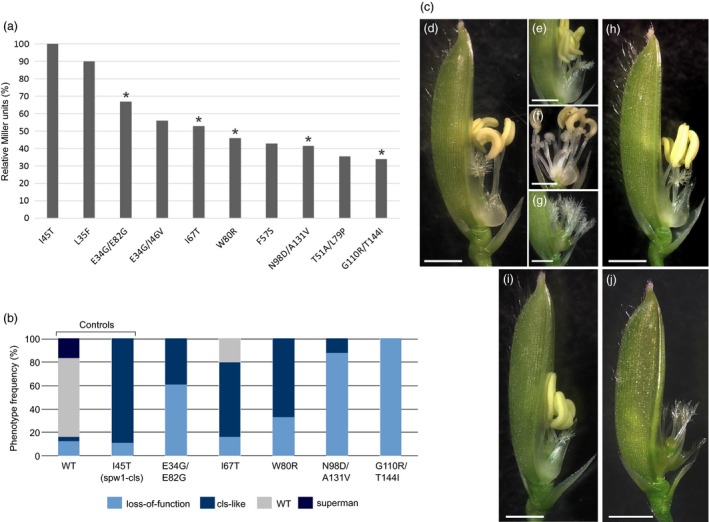
Yeast two‐hybrid and complementation analysis of selected *spw1* mutants. a: β‐Galactosidase activity of selected mutants relative to that of *spw1‐cls* (I45T) in a yeast hybrid assay. Mutations selected for further analysis displayed in b are indicated by asterisks. b: Phenotype frequency in *spw1‐1* lines complemented with selected mutated genomic constructs. c: Characteristic floral phenotypes observed in complementation lines. From (d) to (g): lines complemented with the WT allele showing a WT (d), cls‐like (e), superman (f) or a loss‐of‐function (g) phenotype. Complementation with an I45T (h), W80R (i) or a G110/T144I (j) construct. For all pictures the lemma was removed to allow observation of the inner organs. Bars = 2 mm.

To provide a more precise assessment of the severity of the isolated mutations, binding affinities between the corresponding mutated SPW1 proteins and OsMADS2 were evaluated comparatively to that of SPW1^I45T^ in a liquid β‐galactosidase assay. β‐Galactosidase activities ranged from about 35% to 90% to that of the SPW1^I45T^/OsMADS2 dimer, indicating that the screen was successful in identifying mutations with intermediate destabilising effects (Figure 2a).

To evaluate to what extent the newly identified mutations would actually affect floral development, five mutants spanning the obtained range of β‐galactosidase activities (E34G/E82G; I67T; W80R; N98D/A131V and G110R/T44I) were selected for testing *in planta*. Each of the selected mutation was introduced into the *SPW1* genomic clone (*gSPW1*) and the resulting constructs were used to complement the *spw1‐1* null mutant, along with wild type (WT) and *spw1‐cls* controls. A minimum of 24 transgenic lines were generated and scored for floral organ development for each of the seven constructs.

Control lines transformed with the WT allele displayed four distinct phenotypes: about two‐thirds of the lines showed the expected WT phenotype, and about 17% showed either a cleistogamous (*cls*‐like) phenotype or a loss‐of‐function phenotype, indicative of a partial and lack of complementation, respectively; the remaining 17% of the lines showed a ‘superman’ phenotype, indicative of overexpression of the transgene (Figure 2b, d–g). Similarly, complementation of the loss‐of‐function *spw1‐1* mutant with the *gSPW1* ^*I45T*^ construct resulted in most lines showing the expected *spw1‐cls* phenotype but also in around ten per cent of lines in which complementation had failed, resulting in a loss‐of‐function phenotype. While complementation had failed in several lines, most likely due to artefactual variations of the level of expression of the transgene based on its insertion locus, the majority of transgenic lines showed the expected complementation phenotypes in both control experiments. Based on this observation, it was assumed that the most frequent phenotype obtained for a given mutant was representative of the severity of its mutation. Lines carrying mutations that resulted in the lowest β‐galactosidase activities in the yeast two‐hybrid assay (e.g. G110R/T144I) showed for the most part a loss‐of‐function phenotype and were completely sterile (Figure 2b, j). Lines carrying mutations that resulted in more intermediate β‐galactosidase values (e.g. W80R) were for the most part producing seeds and showing a *cls*‐like phenotype (Figure 2b, i). Thus, mutations resulting in around 50 per cent of β‐galactosidase activity in the yeast two‐hybrid assay are good candidates for genetic engineering. Altogether, these results show that progressive disruption of the heterodimer leads to an increasing loss of lodicule identity, validating our prerequisite for potential fine‐tuning of the SPW1/OsMADS2 heterodimer activity.

### A novel cleistogamous allele of *SPW1* isolated in a forward genetic screen

**Figure 3:**
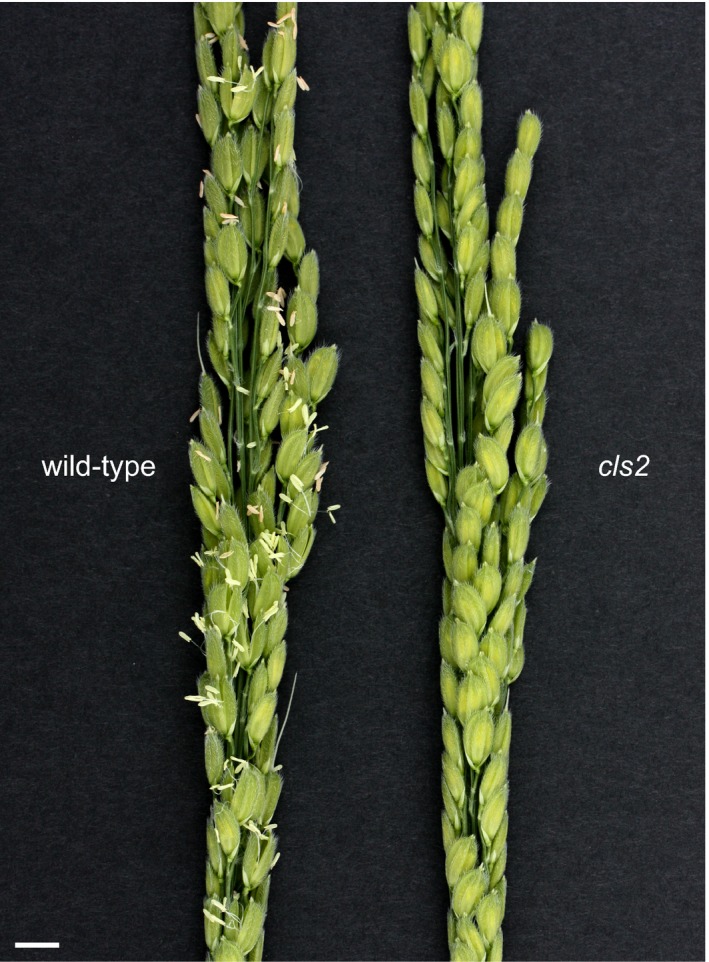
Details of a wild type inflorescence after anthesis compared with a *cls2* mutant inflorescence. Bar = 1 cm.

**Figure 4:**
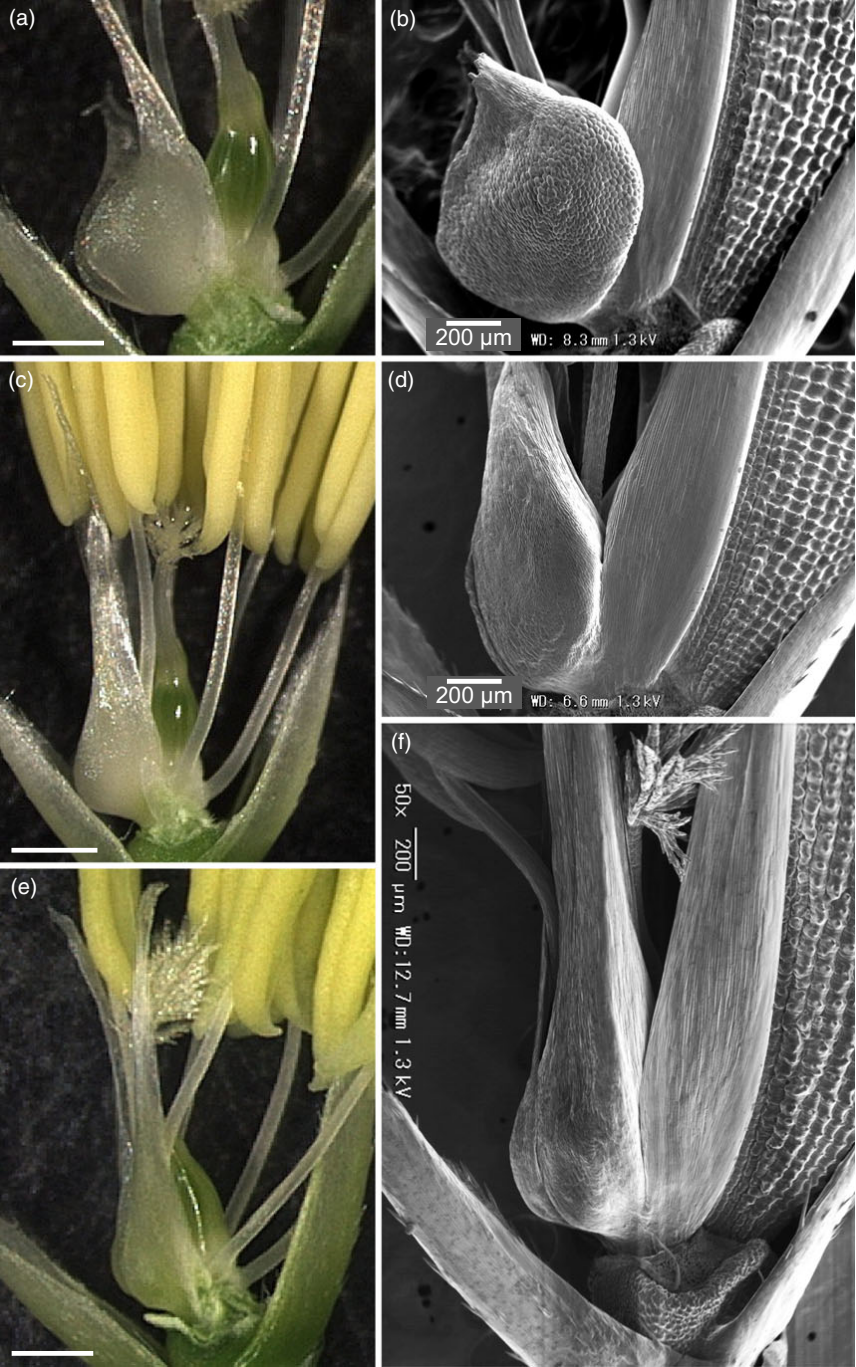
Microscopic observations of lodicules of the *spw1‐cls* and *cls2* mutants. Light and electronic microscopic observations of WT (a, b), *spw1‐cls* (c, d) and *cls2* (e, f) lines. Lemmas were removed for electronic microscopy pictures and both lemma and palea were removed for light microscopy pictures. Bars= 500μm unless indicated otherwise.

**Figure 5:**
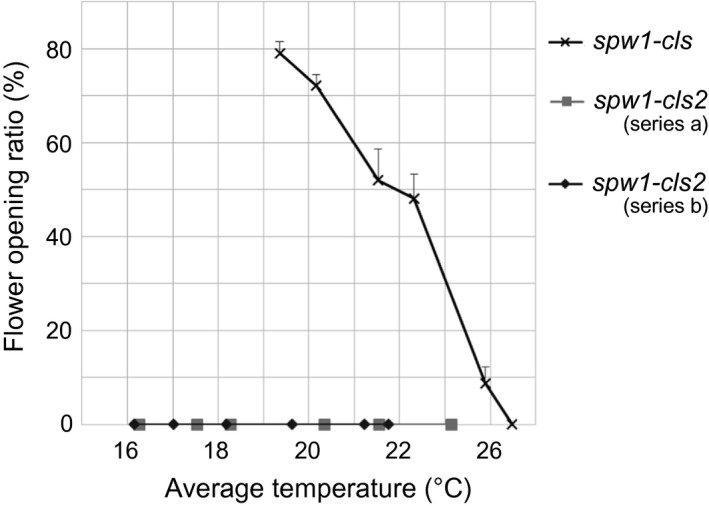
Effect of temperature on flower opening in the *spw1‐cls* and *cls2* mutant lines. Plants were transferred to a temperature‐controlled growth chamber between four to seven weeks before heading. Each series corresponds to a single experiment.

A cleistogamous line, provisionally named *cleistogamy2* (*cls2*), was isolated from a chemically mutagenised rice population (cv Kita‐aoba). In contrast to the WT, stamen exertion could not be seen in *cls2* (Figure 3). Microscopic observations of *cls2* flowers revealed that the lodicules were thin and elongated, resembling the lodicules of the *spw1‐cls* mutant (Figure 4c, e). Furthermore, the surface of the lodicules in *cls2* was populated by both round cells and elongated cells, a pattern also observed in *spw1‐cls*, suggesting a partial loss of lodicule identity (Figure 4d, f). Growth chamber experiments revealed that, unlike for *spw1‐cls*, the cleistogamous phenotype of *cls2* was maintained in a cold environment: for the *spw1‐cls* mutant, open flowers could be seen from temperature averages below 26 °C and about half of *spw1‐cls* flowers were open for averages of 23 °C (Figure 5). In contrast, flowers of the *cls2* mutant remained closed for temperature averages as low as 17 °C.

The *SPW1* and *OsMADS2* genes being strong candidates for the causal gene of the elongated lodicule phenotype, both genomic sequences were checked for mutations in *cls2*. A guanine to adenine transition was found in *SPW1*, leading to a glycine to arginine change in the MADS domain of the protein (G27R; Figure 1). Allelism tests confirmed that *SPW1* was the causal gene of the cleistogamous phenotype in *cls2* (Supplemental Figure 1). The *cls2* and *spw1‐cls* alleles were therefore renamed to *spw1‐cls2* and *spw1‐cls1*, respectively.

To evaluate field performance and agronomic traits of the *spw1‐cls2* line, plants were grown in paddy fields in three locations of Japan: Tsukubamirai, Joetsu and Sapporo, in order of increasingly colder climates. Preliminary data indicate that the fertility of *spw1‐cls2* is negatively affected by low temperatures, with about 90% (Tsukubamirai), 75% (Joetsu) and 65% (Sapporo) of the wild type fertility. Further study in Tsukubamirai showed that *spw1‐cls2* was comparable to the WT for heading date, culm number and length, panicle length and spikelet number (Table 2). In all three locations only rare flower openings were observed, with rates below one per cent at the coldest location of Sapporo, indicating that the cleistogamous phenotype of *spw1‐cls2* is remarkably stable in field conditions.

**Table 2:** Agronomic characteristics of the *spw1‐cls2* line compared with the wild type

	Time to heading (days)	Culm length (cm)	Panicle length (cm)	No. of panicle per plant	No. of spikelet per panicle
*spw1‐cls2*	77	63.9 ± 2.6	18.6 ± 1.0	14.2 ± 1.7	182.0 ± 24.1
Wild type	76	61.5 ± 3.6	18.0 ± 1.0	12.6 ± 1.2	189.7 ± 29.9

Data represent mean values ± standard deviation from 10 plants grown in paddy fields located in Tsukubamirai. Time to heading corresponds to the number of days from seedling to heading.

### The *spw1‐cls2* mutant is impaired for CArG box recognition by the SPW1/OsMADS2 heterodimer

The glycine in position 27 is a conserved residue of the MADS domain (Jeon *et al*., 2000; Silva *et al*., 2016) and has been shown to be involved in binding to DNA target elements called CArG boxes (Schwarz‐Sommer *et al*., 1992). In the *spw1‐cls2* mutant, the G27R change in the SPW1 protein is therefore likely to interfere with its DNA‐binding function.

**Figure 6:**
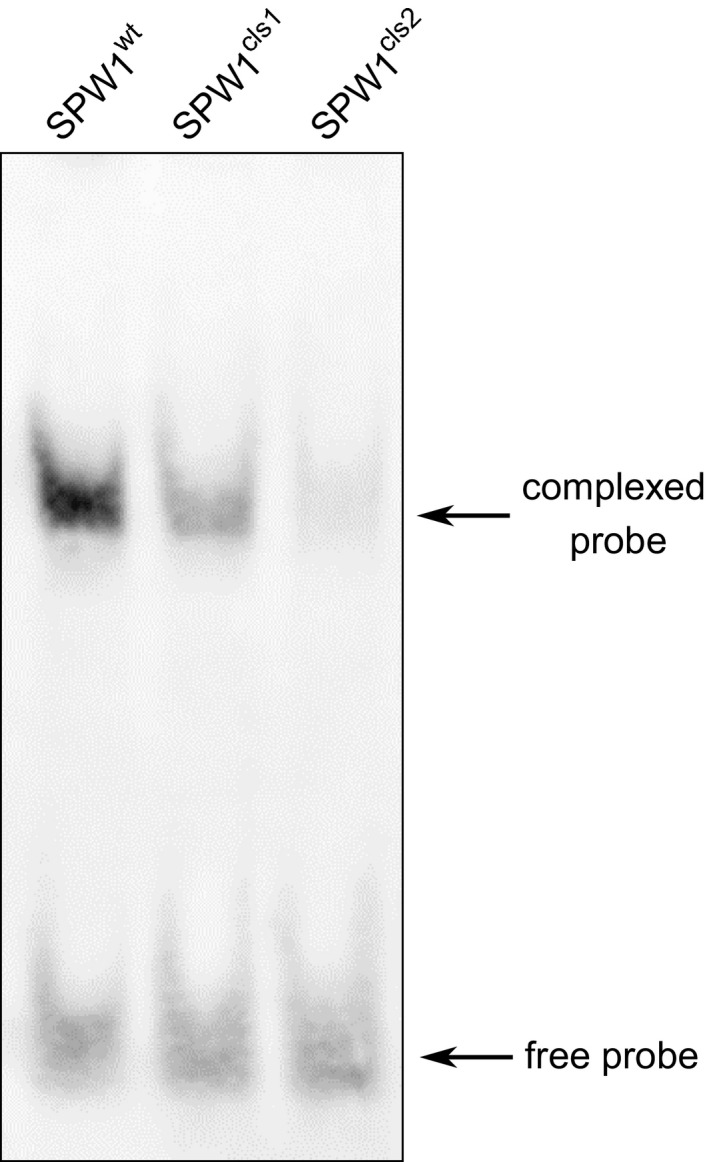
Electrophoretic mobility shift assay between a *DEF* promoter CArG probe and heterodimers of OsMADS2 and SPW1^WT^, SPW1^cls1^ (SPW1^I45T^) or SPW1^cls2^ (SPW1^G27R^).

CArG boxes are variations of a core CC(A/T)_6_GG motif, and different CArG boxes are specifically bound by different MADS‐box protein complexes (Shore and Sharrocks, 1995). CArG loci bound by SPW1 transcriptional complexes have yet to be identified; however, data about *SPW1* and *OsMADS2* orthologs are available. In *Antirrhinum majus, DEFICIENS* (*DEF*) and *GLOBOSA* (GLO) are the respective orthologs of *SPW1* and *OsMADS2*. The DEF/GLO heterodimer has been shown to bind efficiently a CArG box located in the *DEF* promoter region (Schwarz‐Sommer *et al*., 1992; Zachgo *et al*., 1995). Using a probe corresponding to the *DEF* promoter CArG box sequence, heterodimers of OsMADS2 and SPW1^I45T^, SPW1^G27R^ or SPW1^WT^ were compared for their binding capacities in an electrophoretic mobility shift assay (EMSA). A pronounced band shift was observed in the lane loaded with the WT heterodimer, confirming that the *DEF*‐CArG probe is significantly bound by SPW1^WT^/OsMADS2 (Figure 6). In comparison, the band shifted by SPW1^I45T^/OsMADS2 was faint and was even fainter in the SPW1^G27R^/OsMADS2 lane, indicating a weak binding between the probe and both of the mutated dimers. This result supports the idea that a defective binding of SPW1 transcriptional complexes to target CArG box loci is responsible for the mutant phenotypes in *spw1‐cls1* and *spw1‐cls2*, with *spw1‐cls2* being more severely affected.

**Figure 7:**
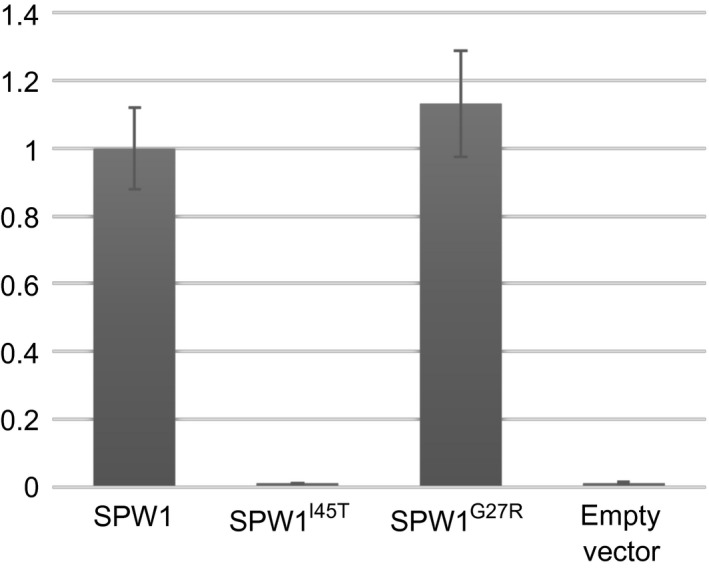
Relative β‐galactosidase activities of SPW1 protein variants and OsMADS2 in a yeast two‐hybrid liquid assay. β‐Galactosidase activities generated in a liquid assay by heterodimers of OsMADS2 and SPW1^I45T^ (*spw1‐cls1*), SPW1^G27R^ (*spw1‐cls2*) relative to that of an OsMADS2/SPW1 (WT) heterodimer was measured at 28 °C.

It was previously demonstrated that the phenotype of *spw1‐cls1* is caused by a destabilisation of the SPW1^I45T^/OsMADS2 dimer (Yoshida *et al*., 2007). The glycine in position 27 of SPW1 sequence does not belong to a region involved in protein dimerisation, and so is not expected to disturb dimer formation (Shore and Sharrocks, 1995; Silva *et al*., 2016). To assess the heterodimerization ability of SPW1/OsMADS2 in *spw1‐cls2*, the binding affinity between SPW1^G27R^ and OsMADS2 was investigated in a yeast two‐hybrid assay. There were no significant differences in β‐galactosidase activities between the SPW1^WT^/OsMADS2 and the SPW1^G27R^/OsMADS2 dimer samples, indicating that, unlike for *spw1‐cls1*, heterodimer formation is not affected in *spw1‐cls2* (Figure 7).

Taken together, the results of the yeast two‐hybrid and EMSA experiments strongly suggest that the *spw1‐cls2* phenotype is caused by a defective binding of transcriptional complexes involving SPW1^G27R^ to target CArG box elements.

## Discussion

### Isolation of an *spw1* cold‐stable cleistogamous line

The ability to prevent cultivated plants from outcrossing with plants of the environment is of particular importance in modern agriculture, all the more so in the light of the environmental concerns expressed by the scientific community and the general public regarding the development and commercialisation of GM crops (Bennett *et al*., 2013). Cultivated rice is mostly self‐pollinating; however, outcrossing with wild relatives is a matter of concern, particularly when plants are grown in vicinity (Marathi and Jena, 2014; Phan *et al*., 2012). Among the various strategies that have been developed to prevent genetic material exchange between cultivated crops and plants of the environment, often collectively referred to as gene containment, cleistogamy has the advantage of strictly preventing cross‐pollination (as opposed to male sterility for example in which flowers are potential pollen recipients) while maintaining plants fertile. In a previous work, it was shown that a single amino acid change in the sequence of the rice transcription factor SPW1 was responsible for the cleistogamous phenotype of the *spw1‐cls1* mutant (Yoshida *et al*., 2007). The one‐base genetic change in *spw1‐cls1* makes the cleistogamous trait of the mutant straightforward to manage from a breeding perspective. Furthermore, *spw1‐cls1* being a non‐transgenic line, its commercialisation would be dispensed with the lengthy and costly regulatory approval processes usually required the in the case of GM material (Mullins, 2014). However, a major drawback of the *spw1‐cls1* line is that a significant number of flowers open when the plants are grown under cool weather (Yoshida *et al*., 2007; Ohmori *et al*., unpublished data). It is thought that low temperatures allow the SPW1/OsMADS2 heterodimer stabilising, resulting in the recovery of the activity of related transcriptional complexes (Yoshida, 2012). Unlike *spw1‐cls1*,* spw1‐cls2* is not impaired for dimerisation with OsMADS2 (Figure 7). The presence of a mutation in the MADS domain of the protein and the observed lower binding affinity to a canonical CArG box probe (Figure 6) strongly suggest that the phenotype in *spw1‐cls2* is caused by a defective binding of SPW1 complexes to physiological DNA targets. Growth chamber experiments revealed that the flowers of *spw1‐cls2* remained closed despite temperature averages as low as 17 °C, contrasting sharply with the phenotype of *spw1‐cls1* (Figure 5). Technical limitations imposed by the growth chamber system did not allow testing for the effects of large amplitude and/or irregular temperature patterns that can typically be observed in fields. Nevertheless, the stability of the cleistogamous trait towards cold in *spw1‐cls2* was confirmed in standard cultivation conditions. Plants grown in fields around the northern city of Sapporo in Japan, which experiences very cool summers, showed only rare (less than one per cent) open flowers.

### Phenotypic fine‐tuning by structural modifications of MADS‐box proteins

Recent structural studies have highlighted the high functional flexibility of the keratin‐like domain of floral MADS‐box proteins (Puranik *et al*., 2014; Silva *et al*., 2016). The critical role of specific amino acids of the keratin‐like domain in the heterodimerization of APETALA3 (AP3) and PISTILLATA (PI), the respective *Arabidopsis thaliana* orthologs of SPW1 and OsMADS2*,* had already been demonstrated in two earlier studies (Yang *et al*., 2003a,b). Numerous evidence indicate that relatively minor amino acid changes affecting multimerization can have significant physiological repercussions, a feature hypothesised to subtend the central role of MADS‐box genes in floral evolution (van Dijk *et al*., 2010). It can be argued that the MADS domain is more evolutionary static while domains involved in protein–protein interactions allow for diversification and specialisation of the regulatory response (Silva *et al*., 2016). Unsurprisingly, modifications of conserved amino acids of the MADS domain often result in severe phenotypes as the transcription factor DNA‐binding function is challenged, as for examples in the *lhs1*,* mfo1‐1* or *soc1* floral mutants (Jeon *et al*., 2000; Lee *et al*., 2008; Ohmori *et al*., 2009). The glycine in position 27 is conserved among MADS‐box proteins and is involved in DNA contact (Pellegrini *et al*., 1995). Several floral mutants altered for G27, like *spw1‐cls2*, have been described in the literature: the *apetala1‐2* and *cauliflower‐3* mutants of *Arabidopsis thaliana* as well as the *def‐nicotianoides* mutant of *Antirrhinum majus* all show a glycine to aspartic acid (G27D) change. Flower development in all these three mutants is severely altered, indicating a loss of function of the mutated proteins (Kempin *et al*., 1995; Mandel *et al*., 1992; Schwarz‐Sommer *et al*., 1992). The isolation of *spw1‐cls2* suggests, however, that mutating any MADS‐box gene as to generate a G27R change would provide with weak alleles, which may be advantageous for phenotypic analysis or crop improvement.

The increasing number of open flowers in *spw1‐cls1* as temperature decreases suggested that there was, at least to some extent, a linear correlation between the stability of SPW1/OsMADS2 transcriptional complexes and lodicule development. In the present study, it was confirmed that destabilisation of the SPW1/OsMADS2 heterodimer (Figure 2a) is associated with hindrance of lodicule development. The range of phenotypes seen in the complementation lines further supports the idea that SPW1/OsMADS2 complexes act quantitatively on target organ development (Figure 2b, c). In addition to the interactions between OMADS2 and OsMADS4, SPW1 has been shown to also interact with OsMADS3, OsMADS6, OsMADS7, OsMADS8, OsMADS14, OsMADS17 and OsMADS58 within different complexes (Lee *et al*., 2003; Seok *et al*., 2010; Yun *et al*., 2013). It is possible that one or more mutants from the random mutagenesis (Figure 2) are also altered for the formation of complexes involving the MADS proteins listed above. As these MADS proteins have been shown to affect floral development to various extents, this could explain why the correlation between the decrease in β‐galactosidase activity (Figure 2a) and the phenotype severity (Figure 2b) of the transgenic plants is not perfect. Altogether, these data indicate a direct correlation between lodicule development and the binding affinity of SPW1 and OsMADS2, suggesting that it is possible to ‘fine‐tune’ the transcription factor complex activity to obtain a desired phenotype. However, despite the availability of a wealth of information on the structural biology of MADS‐box proteins, predicting the steric effects of a given mutation and the associated downstream physiological changes remains extremely challenging. In this work, it was confirmed that assessing the binding affinity between SPW1 and OsMADS2 via yeast two‐hybrid experiments could provide for a quick and fairly reliable indication of the severity of a given mutation towards floral development. Most likely, following a similar strategy to the one outlined in this work, which is originally based on (Yang *et al*., 2003a), would allow identifying weak alleles in MADS‐box genes other than *SPW1*.

Fertility rates are expected to decrease when the SPW1/OsMADS2 dimer activity falls below a certain threshold as stamen development is also dependent on the heterodimer function. Data from fields located in Tsukubamirai showed around a ten per cent reduction in fertility of *spw1‐cls2* compared with that of the wild type. An unexpected result, however, is the further decreased fertility observed in fields located in the colder areas of Joetsu and Sapporo. In the case of *spw1‐cls1*, low temperatures allow restoring the SPW1/OsMADS2 function and consequently lodicule and stamen development. Although mutations in *spw1‐cls1* and *spw1‐cls2* affect the functions of the respective transcription factors via different mechanisms, fertility was not anticipated to be negatively affected by low temperatures in *spw1‐cls2*. It is possible that the decrease in the SPW1/OsMADS2 activity leads to a greater susceptibility to cold stress, however, by which mechanisms this would occur is left to speculation. One solution to maintain high fertility rates might be to mutate the MADS domain of OsMADS2 instead of that of SPW1 as this would maintain SPW1/OsMADS4 activity in the third whorl, which is also supporting stamen development (Yao *et al*., 2008; Yoshida, 2012).

### Concluding remarks

Despite a lower fertility, the *spw1‐cls2* can be advantageously used for specific purposes. Coupled with isolation cultivation practices, *spw1‐cls2* would provide minimal risks of transgene escape and, to the best of our knowledge, has no equivalent to date in rice. The use of *spw1‐cls2* is not limited to transgene containment and rice producers who are particularly inclined to preserving the purity of a cultivar would also benefit from its adoption. As such, the cleistogamous line isolated in this study constitutes a valuable resource for rice cultivation.

## Experimental procedures

### Yeast two‐hybrid assays

The coding sequence of *OsMADS2* as well as the wild type and mutated coding sequences of *SPW1* comprising the M, I and K domains were subcloned into the two‐hybrid vectors pAS2‐1 and pACT2, respectively (Clonetech). All yeast transformations were performed using Mav203 as the host strain as described previously (Yoshida *et al*., 2007). For colorimetric filter assay, yeast cells were incubated at 18 °C to allow for visual detection of weak interactions (Yoshida *et al*., 2007) and *lacZ* reporter gene activity in yeast cells was monitored visually using a 5‐bromo‐4‐chloro‐3‐indolyl‐β‐d‐galactopyranoside (X‐Gal). For quantification of β‐galactosidase activity, yeast cells were grown in liquid cultures to log phase at 25 °C unless otherwise stated and ONPG was used as substrate as described in (Yoshida *et al*., 2007).

### Screening of mutagenised cDNAs of SPW1

Mutated *SPW1* cDNAs were created using error‐prone PCR amplification (Tarun *et al*., 1998) and cloned into the pACT2 vector to generate a library of about 10 000 independent clones, following Clonetech protocol. Clones were screened in a coloration filter assay as described above.

### Plant transformation

Mutations selected from the yeast two‐hybrid assay were introduced into a genomic DNA fragment of *SPW1* and used for complementation of the *spw1‐1* mutant. Mutated and WT control DNA fragments were cloned into the pZH2B vector (Kuroda *et al*., 2010). Resulting vectors were introduced into scutellum‐derived calli of *spw1‐1* by *Agrobacterium*‐mediated transformation under selection with hygromycin, as described in (Oikawa *et al*., 2004). The genetic background of *spw1‐1* is the cultivar ‘Kinmaze’.

### Identification of the *spw1‐cls2* mutant

To identify cleistogamous mutants of rice, an M_2_ population of *Oryza sativa* L. ssp. *japonica* cv. Kita‐aoba mutagenised with ethyl methanesulfonate was screened. The plants were grown in a paddy field of the NARO Hokkaido Agricultural Research Center (Sapporo, Japan, formerly the National Agricultural Research Center for Hokkaido Region) under standard conditions. Cleistogamous plants were selected following the method described by Yoshida *et al*. (2007) and subsequent examination revealed one promising line in which cleistogamy was inherited as a single recessive trait.

### Temperature gradient growth chamber experiments

Plants of *spw1‐cls1* and *spw1‐cls2* were grown in a natural lit chamber at the NARO Tohoku Agricultural Research Center (Morioka, Japan) with a 26 °C/20 °C day/night temperature regime. A minimum of 15 seeds were sown in three‐litre circular plastic pots with 0.9 g each of N, P_2_O_5_ and K_2_O. At panicle formation stage, pots were transferred to a temperature gradient chamber. Six pots of each line were placed in different positions along the temperature gradient. Open flowers were scored and marked every two to three days and the total number of flowers was counted after full maturation.

### Agronomic traits of *spw1‐cls2*

To evaluate the agronomic characteristics of *spw1‐cls2*, including flower opening rate and fertility, plants were cultivated under standard conditions in paddy fields of the NARO Institute of Crop Science (Tsukubamirai, Ibaraki, JAPAN), NARO Agricultural Research Center (Joetsu, Niigata, JAPAN) and NARO Hokkaido Agricultural Research Center (Sapporo, Hokkaido, JAPAN) during summers of 2011 and 2012.

### Electrophoretic mobility shift assay

For each protein, the corresponding full‐length CDS was cloned into a pF3A‐WG expression vector (Promega). Each one of the three (SPW1, spw1‐cls and spw1‐cls2) proteins was co‐synthesised with OsMADS2 using a TNT SP6 High‐Yield wheat germ protein expression system (Promega) according to the manufacturer's instructions. A synthetic probe corresponding to a *DEFICIENS* CArG box (5′‐GGCAACTCTTTCCTTTTTAGGTCGCATATGG‐3′) was labelled with digoxigenin using a DIG gel shift kit reagents (Roche) and 100 fmol of the probe was treated with 0.03 units of proteinase K (Sigma P2308) for 30 min at 37 °C followed by a 60‐min deactivation at 60 °C to remove DIG transferase contamination from the probe sample. A total of about 1 μg of protein was put in presence of 20 fmol of labelled probe for 20 min at 25 °C in a 20 μL binding solution containing 2.5% glycerol, 5 mm MgCl_2_, 50 ng Poly (dI·dC) and 0.05% NP‐40 and 1X binding buffer (Thermo Scientific 20148A). Samples were loaded on a 6% polyacrylamide gel and detected after blotting in a chemiluminescent reaction using a LAS‐3000 imager (Fujifilm).

## Supporting information


**Figure S1** Spikelet of the F1 progeny of a *spw1‐cls* and *cls2* cross. The lemma has been removed to allow observation of the inner organs. Bar = 2 mm.
