# Supplementary figures and images for: The superwoman1‐cleistogamy2 mutant is a novel resource for gene containment in rice
Source: Plant Biotechnol J. 2016 Jul 18;15(1):97–106. doi: 10.1111/pbi.12594 (PMC5253472; doi:10.1111/pbi.12594)

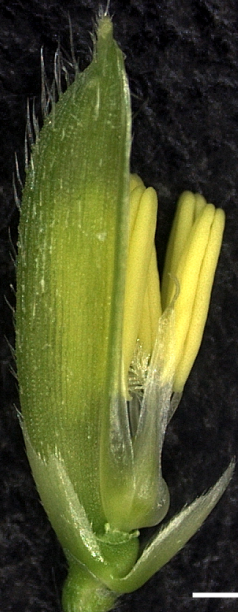

Supplement: Supplementary file 1 — Figure S1 Spikelet of the F1 progeny of a spw1‐cls and cls2 cross. The lemma has been removed to allow observation of the inner organs. Bar = 2 mm. [file PBI-15-97-s001.pdf]
